# Cardiogenic Pulmonary Edema in Emergency Medicine

**DOI:** 10.3390/arm91050034

**Published:** 2023-10-13

**Authors:** Christian Zanza, Francesco Saglietti, Manfredi Tesauro, Yaroslava Longhitano, Gabriele Savioli, Mario Giosuè Balzanelli, Tatsiana Romenskaya, Luigi Cofone, Ivano Pindinello, Giulia Racca, Fabrizio Racca

**Affiliations:** 1 Post Graduate School of Geriatric Medicine, University of Rome “Tor Vergata”, 00133 Rome, Italy; 2 Italian Society of Prehospital Emergency Medicine (SIS 118), 74121 Taranto, Italy; 3 Department of Emergency and Critical Care, Santa Croce and Carle Hospital, 12100 Cuneo, Italy; 4 Department of Systems Medicine, University of Rome “Tor Vergata”, 00133 Rome, Italy; 5 Department of Anesthesiology and Perioperative Medicine, University of Pittsburgh, Pittsburgh, PA 15261, USA; 6 Department of Emergency Medicine, Humanitas University Hospital, 20089 Rozzano, Italy; 7 Emergency Department, IRCCS Fondazione Policlinico San Matteo, 27100 Pavia, Italy; gabrielesavioli@gmail.com; 8 Department of Physiology and Pharmacology, Sapienza University of Rome, 00185 Rome, Italy; 9 Department of Public Health and Infectious Diseases, Sapienza University of Rome, 00185 Rome, Italy; luigi.cofone@uniroma1.it (L.C.); ivano.pindinello@uniroma1.it (I.P.); 10 Division of Anesthesia and Critical Care Medicine, AO Ordine Mauriziano, 10128 Turin, Italy; giulia.racca2000@gmail.com (G.R.);

**Keywords:** cardiogenic pulmonary edema, capillary permeability, pulmonary surfactant-associated protein B, non-invasive ventilation

## Abstract

**Highlights:**

**What are the main findings?**

- Cardiogenic pulmonary edema is the most common cause of respiratory failure and results from increased cardiac filling pressure and alveolar-epithelial barrier breakdown due to factors like inflammation, leukocyte infiltration, procoagulant processes, and ion channel modification by reactive oxygen and nitrogen species.
- Rapid evaluation of the patient with cardiogenic pulmonary edema and appropriate treatment are necessary to reduce mortality and morbidity.

**What is the implication of the main finding?**

- Bedside tests, especially ultrasound, help us to quickly identify and to understand the need of amine support. Optimal therapy for patients with cardiogenic pulmonary edema hinges on precise phenotypic identification and etiological assessment, with early consideration of non-invasive ventilation and diuretics.
- Hypoperfusion necessitates inotropes and, at times, vasopressors, while individuals exhibiting persistent symptoms and diuretic resistance may benefit from vasodilators and additional therapeutic strategies, including beta-agonists, underscoring the need for further studies to explore medical interventions in mitigating pulmonary damage in CPE patients.

**Abstract:**

Cardiogenic pulmonary edema (CPE) is characterized by the development of acute respiratory failure associated with the accumulation of fluid in the lung’s alveolar spaces due to an elevated cardiac filling pressure. All cardiac diseases, characterized by an increasing pressure in the left side of the heart, can cause CPE. High capillary pressure for an extended period can also cause barrier disruption, which implies increased permeability and fluid transfer into the alveoli, leading to edema and atelectasis. The breakdown of the alveolar-epithelial barrier is a consequence of multiple factors that include dysregulated inflammation, intense leukocyte infiltration, activation of procoagulant processes, cell death, and mechanical stretch. Reactive oxygen and nitrogen species (RONS) can modify or damage ion channels, such as epithelial sodium channels, which alters fluid balance. Some studies claim that these patients may have higher levels of surfactant protein B in the bloodstream. The correct approach to patients with CPE should include a detailed medical history and a physical examination to evaluate signs and symptoms of CPE as well as potential causes. Second-level diagnostic tests, such as pulmonary ultrasound, natriuretic peptide level, chest radiograph, and echocardiogram, should occur in the meantime. The identification of the specific CPE phenotype is essential to set the most appropriate therapy for these patients. Non-invasive ventilation (NIV) should be considered early in the treatment of this disease. Diuretics and vasodilators are used for pulmonary congestion. Hypoperfusion requires treatment with inotropes and occasionally vasopressors. Patients with persistent symptoms and diuretic resistance might benefit from additional approaches (i.e., beta-agonists and pentoxifylline). This paper reviews the pathophysiology, clinical presentation, and management of CPE.

## 1. Introduction

Cardiogenic pulmonary edema (CPE) can be a life-threatening condition leading to compromised gas exchange and acute respiratory failure (ARF) associated with high mortality. Without prompt recognition and treatment, a severe form can deteriorate rapidly, resulting in severe hypoxia [1,2]. Prognosis mainly depends on the underlying medical conditions, but generally, these patients have a poor prognosis with an in-hospital death rate of 15–20%, a rate of survival of 50% after one year [3], and a mortality rate of 85% at 6 years follow-up [4].

CPE is defined as pulmonary edema caused by the accumulation of excessive fluid in the lung’s interstitial and alveolar spaces due to increased capillary hydrostatic pressure secondary to elevated pulmonary venous pressure, which is the result of elevated cardiac filling pressures [5]. All the cardiac diseases that contribute to increased pressure in the left side of the heart can cause CPE [6]. However, there are many other clinical conditions of non-cardiological origin (i.e., primary fluid overload, severe hypertension, and severe renal disease) that may be responsible for CPE causing elevated cardiac filling pressures without heart disease. Noncardiogenic pulmonary edema (NCPE) is a distinct form of pulmonary edema caused by increased permeability of the pulmonary endothelium without elevated capillary hydrostatic pressure. The most frequent cause of NCPE (also called Adult Respiratory Distress Syndrome (ARDS)) is due to direct (e.g., pneumonia) or indirect lung injury (e.g., sepsis). Increased alveolar-capillary permeability may also occur in CPE, especially in some patients with abrupt, severe increases in pulmonary capillary pressure [7]. This associated barrier dysfunction was defined as “stress failure” [8] and may lead to more edema formation [9].

Rapid identification of the specific heart failure (HF) is essential to set up the appropriate therapy. Diuretics are considered the cornerstone of CPE treatment [10], and non-invasive ventilation (NIV) should be considered in an early stage [11]. Vasodilators may also be required to decrease elevated filling pressures and/or left ventricle (LV) afterload. In addition, patients with persistent symptoms might benefit from additional approaches, including beta-agonists and pentoxifylline, which may be effective in patients with “stress failure” [9]. Elevated serum levels of surfactant protein B levels could be used to identify CPE patients with associated barrier damage [12].

This paper reviews the pathophysiology and management of CPE.

## 2. Pathophysiology of Pulmonary Edema

Pulmonary edema refers to excessive fluid accumulation in the interstitial and alveolar spaces of the lungs due to an imbalance among the “Starling forces”, an increase of alveolar-capillary barrier permeability, or a blockage of the draining lymphatic system. [6].

Fluid balance between the interstitium and vascular bed in the lung, as in other microcirculations, is determined by the Starling relationship, which predicts the net flow of liquid across a membrane. This is represented in the following equation: Net filtration = Kf × (Δhydrostatic pressure − Δoncotic pressure), where Kf is the filtration coefficient.

In normal circumstances, the pulmonary capillary hydrostatic pressure ranges from 6 to 13 mmHg. The oncotic pressure is determined by proteins in human plasma; the normal mean value is 21.1 mmHg in those younger than 50 years old and significantly lower at 19.7 mmHg in those between ages 70 and 89. The interstitium is generally kept “dry” by the balance of these forces. Moreover, an initial rise in the amount of fluid moving from the pulmonary capillaries to the interstitial spaces can be balanced by the lymphatic system.

**Figure 1:**
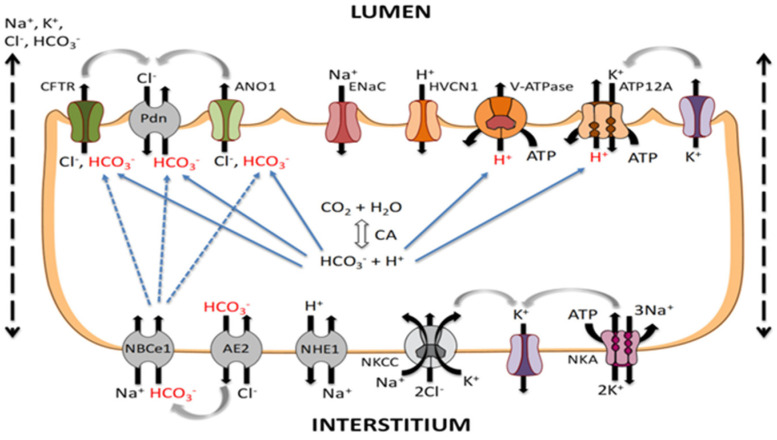
Ion transport across alveolar epithelial cells. Na^+^ and Cl^−^ ions enter the apical membranes (lumen) of alveolar epithelial cells (both Type I and Type II) via Epithelial sodium channels (ENaC) and Cl^−^ channels such as cystic fibrosis transmembrane conductance regulator (CFTR) located in the apical face of the cell (interstitium). The Na^+^ ions are driven by an electrochemical gradient created by the energy-dependent Na/K-ATPase. K^+^ ions, brought in by the Na/K-ATPase pump in exchange for the Na^+^ ions, leave the cell passively through K^+^ channels located in the basolateral membrane. CFTR: Cystic fibrosis transport regulator; ENaC: Epithelial sodium channel.

In a healthy lung, the alveolar epithelium is a tight barrier that prevents the passage of water, electrolytes, and hydrophilic solutes to the air spaces; likewise, the capillary endothelium constitutes a semipermeable barrier to fluid exchange [13]. To avoid a build-up of fluid in the lungs, the alveolar epithelial cells actively transfer sodium (Na) and chloride (Cl) ions. Through sodium-selective cation channels and cycling nucleotide-ungated channels, both Type I and Type II alveolar epithelial cells take in Na ions, which are then extruded across the basolateral membrane by an energy-dependent Na/K- ATPase [14]. This vectorial transport of Na ions and the concomitant movement of Cl ions leads to the reabsorption of fluid across both normal and damaged lungs. Figure 1 shows the ion transport between alveolar epithelial cells, which is essential for maintaining the fluid balance.

Pulmonary stress, either via direct damage or mediated by Reactive Oxygen And Nitrogen Species (RONS), can modulate the activity of these channels [14,15,16]. Indeed, one possible etiology of alveolar permeability to plasma proteins is oxidant injury. In this case, RONS can modify or damage epithelial sodium channels, altering fluid balance.

In patients with CPE, an increase in pulmonary venous and left atrial (LA) pressure, which most frequently occurs from an elevated LV filling pressure, causes the rise in pulmonary capillary of the hydrostatic pressure. According to the Starling law, the pressure must increase higher than the normal value of the plasma colloid osmotic pressure for edema to occur [12,17,18]. The functional capacity of the lymphatic system to remove the extra fluid is different from patient to patient and with the severity of the disease. These characteristics influence the rate of accumulation of lung fluid at a given elevation in pulmonary capillary pressure [19]. An acute rise in pulmonary arterial capillary pressure (i.e., to >18 mm Hg) may increase fluid filtration into the lung interstitium, but the lymphatic removal does not increase correspondingly. As a result, pulmonary edema may occur at pulmonary capillary pressures as low as 18 mmHg. In contrast, in the presence of chronically elevated LA pressure, the rate of lymphatic removal can be as high as 200 mL/h, which protects the lungs from CPE. Thus, these patients do not develop CPE until significantly higher pulmonary capillary pressures are reached.

**Figure 2:**
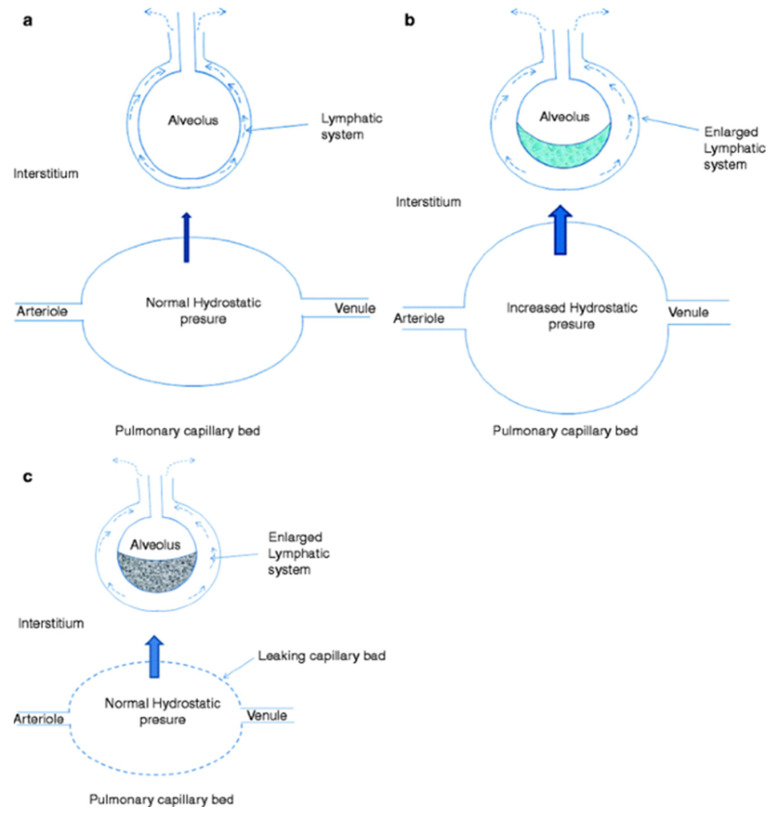
Physiology of Microvascular Fluid Exchange in the Lung. In the normal lung (**a**), fluid moves continuously outward from the vascular to the interstitial space according to the net difference between hydrostatic and protein osmotic pressures, as well as to the permeability of the capillary membrane, according to the Starling equation. When hydrostatic pressure increases in the microcirculation (cardiogenic pulmonary edema), the rate of transvascular fluid filtration rises (**b**). Noncardiogenic pulmonary edema (**c**) occurs when the permeability of the microvascular membrane increases because of direct or indirect lung injury (including acute respiratory distress syndrome), resulting in a marked increase in the amount of fluid and protein leaving the vascular space. Noncardiogenic pulmonary edema has a high protein content because the more permeable microvascular membrane has a reduced capacity to restrict the outward movement of larger molecules such as plasma proteins.

The most common cause of NCPE (i.e., ARDS) is characterized by the presence of the injury of the alveolar-capillary barrier in the absence of elevated pulmonary capillary pressures. It is caused by direct (e.g., pneumonia) or indirect lung injuries (e.g., sepsis) (Figure 2). Other conditions that may cause NCPE include decreased oncotic pressure of the plasma (caused by hypoalbuminemia), increased negativity of interstitial pressure (e.g., rapid removal of pneumothorax), and lymphatic insufficiency (e.g., from lymphangitic carcinomatosis, fibrosing lymphangitis, or lung transplantation). Hypoalbuminemia occurs in several diseases, including malabsorption and malnutrition, renal disease, where the loss of albumin occurs across the glomerulus (e.g., nephrotic syndrome), or hepatic diseases, where there is inadequate albumin synthesis.

Although CPE usually occurs in the absence of change in the permeability of the alveolar-capillary barrier [20], the capillary wall’s permeability may also be affected during CPE. A sudden increase in pulmonary capillary hydrostatic pressure (i.e., flash pulmonary edema) [7] can cause mechanical damage on the alveoli–capillary barrier through a process known as “stress failure” [8]. Renal artery stenosis, particularly when bilateral, has been identified as a common cause of flash pulmonary edema [7]. In case of stress failure, both hydrostatic pressures and raised permeability increase fluid transfer into alveolar spaces. Finally, inflammation associated with stress failure may cause surfactant alterations [7,8,21]. As a result, there is an increase in permeability due to the loss of tight junction-mediated cell-to-cell contact, changes in extracellular matrix (ECM), and surfactant dysfunction. This acute tissue injury is also characterized by the over-activation of leukocytes and platelets and a dysregulated inflammatory response with an increased level of tumor necrosis factor-alpha (TNF-alpha), synthesized by macrophages, endothelial cells, and alveolar type II epithelial cells [22,23]. In addition, in patients presenting with CPE associated with stress failure, surfactant protein levels in the serum (i.e., surfactant protein-A and surfactant protein-B) may be elevated [12].

From a molecular point of view, another important aspect to consider is the downregulation of the epithelial Sodium channels (ENaC) due to hypoxia and hypercapnia [24,25]. In addition, cell necrosis and fluid accumulation could trigger an inflammatory response. Pro-apoptotic cellular factors, including Bax, Caspase-3, and p53, are activated in the lung along with the apoptotic alterations, as can be seen with a bronchoalveolar lavage (BAL) [26,27]. Another important mechanism underlying pulmonary edema is the activation of the pro-apoptotic Fas/FasL pathway. It has been demonstrated that blocking the activation of this pathway may reduce alveolar damage and the formation of pulmonary edema [28,29]. Several studies show that cytokines, including interleukin (IL)-1β, IL-8, and TNF-α, are involved in alveolar injury and the reduction of alveolar fluid clearance (AFC) [30,31,32]. Further studies are needed to investigate the pathophysiology of alveoli–capillary barrier mechanical injury, which may contribute to the development and maintenance of pulmonary edema in some patients with CPE.

Pulmonary edema leads to progressive deterioration of alveolar gas exchange and respiratory failure. The pathophysiology of respiratory failure includes an increase in extravascular lung water, a reduction in pulmonary compliance, and an increase in airway resistance. These events result in increased work breathing. Moreover, they also cause changes in the ventilation-perfusion ratio and in intrapulmonary shunting, which lead to arterial hypoxemia. Finally, increased work of breathing may induce rapid shallow breathing, leading to hypercapnia.

## 3. Etiology of Cardiogenic Pulmonary Edema

Patients with CPE represent a heterogeneous population. CPE is most commonly due to left cardiac dysfunction. However, a variety of other conditions may promote the development of CPE, causing elevated cardiac filling pressures even without heart disease. They include primary fluid overload, severe hypertension (particularly renovascular hypertension), and severe renal disease. In patients with hemodialysis-dependent renal failure, CPE is often the result of noncompliance with dietary restrictions or with hemodialysis sessions.

Left cardiac dysfunction resulting in CPE includes the following conditions: (i) systolic heart failure, (ii) diastolic heart failure, (iii) heart failure related to left-sided valvular disease, and (iv) heart failure related to arrhythmias [33].

Approximately 50 percent of patients hospitalized for HF are characterized by a preserved left ventricular ejection fraction (LVEF) (LVEF > 50 percent); the remaining patients have HF with reduced ejection fraction (LVEF ≤ 40 percent) or HF with mid-range ejection fraction (LVEF 41 to 50 percent).

Systolic heart failure is characterized by reduced LVEF. Common causes of reduced LVEF include coronary artery disease, long-standing hypertension, idiopathic dilated cardiomyopathy, viral myocarditis, progressive valvular heart disease (i.e., aortic and mitral regurgitation, aortic stenosis), takotsubo cardiomyopathy and toxins such as alcohol, cocaine, and chemotherapeutic agents (Table 1).

The cardiotoxic effects of the anthracyclines are not completely understood. The most widely accepted theory is the formation of anthracycline–iron complexes, which stimulate free-radical formation. The pathogenesis of trastuzumab cardiotoxicity appears to be related to blockage of Human Epidermal Growth Factor Receptor 2 (HER2) signals in cardiac myocytes, which are essential for cardiac myocyte repair [34]. Cisplatin can cause severe high blood pressure, which can lead to heart problems.

Diastolic dysfunction refers to an increase in ventricular stiffness and impaired relaxation, which prevents ventricular filling during diastole. Due to this decreased compliance, a heightened diastolic pressure is required to achieve a similar stroke volume. The LVEF is often preserved. Despite normal LV contractility, cardiac output may be reduced.

The most common causes of diastolic dysfunction are chronic disorders, such as hypertension and left ventricular hypertrophy. Other causes include hypertrophic cardiomyopathy and infiltrative diseases (i.e., sarcoidosis and amyloidosis). Diastolic abnormalities can also be caused by constrictive pericarditis and tamponade. Myocardial ischemia can acutely worsen diastolic dysfunction and cause systolic dysfunction. With a similar mechanism, myocardial contusion induces systolic and diastolic dysfunction.

Left-sided valvular disease that can cause CPE include both stenosis and regurgitation. Critical aortic stenosis results in chronic LV outflow obstruction associated with LV hypertrophy, which decreases ventricular compliance and can eventually cause diastolic and systolic dysfunction. The chronic obstruction to atrial outflow due to mitral stenosis leads to elevated left atrial pressure. Patients with mild or moderate mitral stenosis are frequently asymptomatic due to the slow progression of this condition, which allows patients to adapt to this pathology. However, conditions that cause an elevated heart rate and decreased diastolic filling time, such as exercise or atrial fibrillation with rapid ventricular response, can result in abrupt elevations of left atrial pressures and CPE. Both chronic and acute aortic or mitral insufficiency cause LV volume overload, which raises LV end-diastolic pressure and LA pressure that may result in CPE. Ventricular septal rupture may cause CPE by the same mechanism.

Tachyarrhythmias may promote the development of CPE. New-onset rapid atrial fibrillation and ventricular tachycardia can be responsible for CPE decreasing diastolic filling time, which may lead to an abrupt elevation of LA pressure.

While the largest proportion of patients with CPE are admitted due to worsening chronic HF, others present with HF for the first time. Decompensation of chronic HF can occur without known precipitant factors but more often occurs with one or more factors. Several precipitating factors can coexist and interact with one another, exacerbating the episode of CPE [35]. In Table 2 are listed the major precipitant factors. Major causes of new-onset CPE are reported in Table 3.

## 4. Clinical Manifestations and Diagnostic Process

Clinical presentations of acute decompensated HF can be categorized according to the adequacy of peripheral perfusion (“warm” versus “cold”) and the presence of congestion (“dry” or “wet”) [36]. Such categorization predicts prognosis and facilitates clinical management. A minority of patients with acute decompensated HF (less than 20 percent) present with a “cold and wet” clinical profile characterized by CPE associated with cardiogenic shock. This category of patients has a higher risk of death.

### 4.1. History and Physical Examination

Many patients with chronic HF report that symptoms have progressively worsened over the previous days and weeks. In a small percentage of patients, an episode of CPE may be the first presentation of HF.

The most frequent clinical manifestations are dyspnoea and profuse diaphoresis. Cough is a common complaint and could be an early clue to worsening pulmonary edema. Pink, frothy sputum may be present in patients with severe disease. Other signs of severe hypoxemia may include neurologic symptoms such as confusion, anxiety, and disorientation. Change in mental status can also be caused by hypercapnia.

In CPE patients’ tachypnoea, tachycardia, and jugular venous distention are frequent physical findings. Patients may be sitting upright and usually appear anxious and diaphoretic with cyanosis of the lips. Tachypnoea may be associated with the use of auxiliary breathing muscles. Auscultation of the lungs generally reveals fine, crepitant rales, but wheezes may also be present. Rales are usually heard at the bases first, and they progress to the apices as the condition worsens. An absence of rales does not imply a lack of pulmonary venous pressure elevation. Diminished air entry at the lung bases is usually caused by a pleural effusion, which is often more frequent in the right pleural cavity than in the left. Although cardiac auscultation may be challenging in an acute scenario, when valvular abnormalities result in PCE, murmurs may be audible. Auscultation of murmurs can help in the diagnosis of acute valvular disorders manifesting with CPE.

The initial detection of hypoxia and the patient’s response to further oxygenation and other treatments are both identified using pulse oximetry before treatment. Oxygen saturation in ambient air is usually less than 90 percent [37].

Hypertension is often present due to the hyperadrenergic state. However, when the blood pressure is found to be significantly elevated, it is more likely to be an important contributing factor to pulmonary edema rather than the consequence of the condition.

If hypotension prevails, this could indicate severe left ventricular systolic dysfunction and cardiogenic shock must be considered. Low perfusion is also characterized by a decline in urine output, cold extremities, skin pallor or mottling, and decreased cognitive functioning. Skin mottling at presentation is an independent predictor of an increased risk of in-hospital mortality [36,37].

Hepatomegaly, hepatojugular reflux, ascites, and peripheral edema may be found in patients with concomitant right ventricular failure. In patients with chronic heart failure, peripheral and pulmonary edema frequently coexist. Leg edema is frequently evident in both legs, particularly in the pretibial region and ankles in ambulatory patients. Sacral and upper thigh edema can be detected in patients who are bedridden.

### 4.2. Tests Available for Diagnosing Pulmonary Edema

The diagnosis of CPE is based primarily on signs and symptoms and supported by appropriate investigations. No single definitive test is available for diagnosing CPE, but clinically, one proceeds from simple to more complex tests while searching for the diagnosis and the associated etiology [37].

Initial testing includes a 12-lead electrocardiogram (ECG), chest X-ray, lung ultrasonography, and laboratory tests comprising arterial blood gas (ABG) and cardiac enzymes. Subsequently, a bedside echocardiogram is extremely important in determining the etiology of CPE. A Brain natriuretic peptide (BNP) level should be obtained when the diagnosis of HF is uncertain.

**Figure 3:**
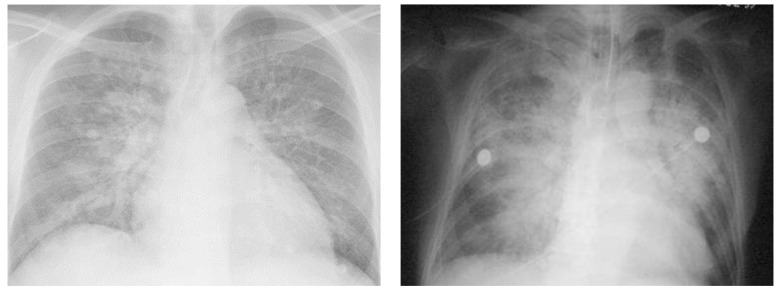
Chest Radiographs representing Cardiogenic and Noncardiogenic Pulmonary Edema. Left X-ray shows an anteroposterior chest Rx with acute anterior myocardial infarction and acute cardiogenic pulmonary edema. Signs of cardiogenic pulmonary edema are peri broncho vascular spaces and the prominent septal lines (Kerleyșs B lines). Right X-ray shows an anteroposterior chest Rx with noncardiogenic pulmonary edema, with blood culture positive for Streptococcus pneumoniae, causing pneumonia complicated by septic shock and acute respiratory distress syndrome. Diffuse alveolar infiltrates appear patchy and bilateral with air bronchograms, findings that are charac-teristics of, but not specific to, noncardiogenic edema and acute lung injury.

Chest radiography is one of the most important investigations required for the evaluation of pulmonary edema and can help distinguish between CPE and NCPE [36,38,39]. However, the characteristic features (Table 4 and Figure 3) described in chest X-rays have only moderate specificity (75–83%) and poor sensitivity (50–68%) in the diagnosis of CPE [38].

**Figure 4:**
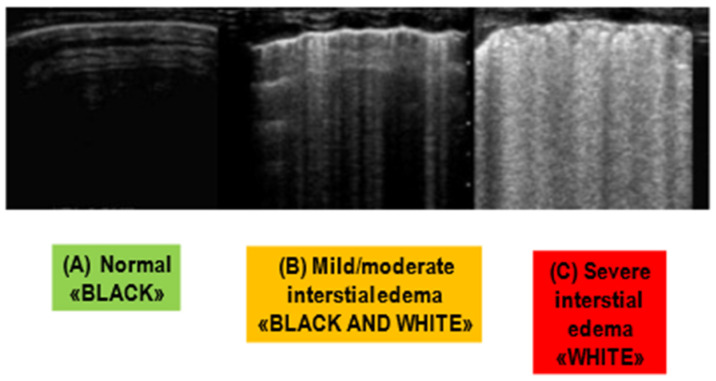
Lung Ultrasounds (LUS). In the normal state (**A**), there are no B-lines. In a state of mild to moderate interstitial edema (**B**), an overall hypoechoic appearance is maintained, with sparse B-lines forming. As the edema progresses to become more clinically severe (**C**), the hypoechoic appearance is lost, with a high density of B-lines.

Transthoracic Pulmonary ultrasound can detect CPE by identifying B-lines, also known as “comet tails,” which represent alveolar-interstitial edema [9]. B-lines are vertical hyperechoic reverberation artifacts that arise at the pleural line and extend to the bottom of the screen (see Figure 4). The extent of edema is correlated to the number of B-lines. In particular, the number of B-lines correlated with extravascular lung water and wedge pressures [40]. In addition, it may predict BNP levels greater than 400 pg/mL and adverse outcomes [41]. Moreover, ultrasound scans reveal whether pleural effusions are present and whether the inferior vena cava collapses, which is a reliable sign of fluid overload. Limitations of this modality include technique dependence and limited specificity in the identification of pulmonary edema [42]. Indeed, a variety of conditions, including pulmonary fibrosis, interstitial pneumonia, pneumonitis, and NCPE, may cause diffuse bilateral B-lines.

Echocardiography is recommended to evaluate LV systolic and diastolic function. Among patients with chronic HF, management strategies differ according to whether or not the patient has HF with reduced LVEF (i.e., LVEF ≤ 40 percent) versus HF with preserved LVEF. Echocardiography is also recommended to evaluate valvular function and to assess for pericardial disease. In addition, new wall-motion abnormalities may suggest acute myocardial infarction.

BNP is a cardiac neurohormone that is secreted from the ventricles when they are under increased pressure and stress. This is one of the HF’s compensatory mechanisms; particularly, it results in renal sodium and water excretion and in the reduction of systemic vascular resistance. Several observational studies and clinical trials have shown the important diagnostic value of BNP measurements in differentiating HF from other pulmonary causes of dyspnoea [43]. Most dyspneic patients with HF have plasma BNP values above 400 pg/mL. The test is less reliable in cases of moderate BNP levels (approximately 200 to 400 pg/mL). Indeed, many conditions (i.e., renal dysfunction, sepsis, COPD, pulmonary embolism, and atrial fibrillation) significantly increase BNP levels. On the other hand, low levels of BNP (<100 pg/mL) usually rule out CPE, although low values are occasionally found in some patients with dyspnoea associated with HF [35].

Pulmonary arterial catheterization using a Swan-Ganz catheter helps in differentiating CPE from NCPE: pulmonary capillary wedge pressure (PCWP) is generally >18 mm Hg in CPE and <18 mm Hg in NCPE. However, routine use of Swan-Ganz catheter in patients with CPE is not required. It is recommended only for patients with CPE with persistent symptoms and/or when the diagnosis is uncertain [44,45].

## 5. Treatment

The initial treatment of CPE is described in Table 5 [44].

### 5.1. Supplemental Oxygen and Assisted Ventilation

Supplemental oxygen therapy and assisted ventilation should be provided as needed to treat hypoxemia (SpO2 < 90 percent). However, oxygen is not recommended as routine therapy in patients without hypoxemia, as it may cause vasoconstriction and reduction in cardiac output [46,47,48].

In case of respiratory distress, respiratory acidosis, and/or hypoxia persists on oxygen therapy, a trial of non-invasive ventilation (NIV) (i.e., continuous positive airway pressure (CPAP) or bilevel NIV) is recommended if there are no contraindications to NIV. This approach is supported by evidence from meta-analyses and small randomized trials in patients with CPE, indicating that NIV improves clinical and laboratory indices of ARF (e.g., heart rate, dyspnoea, hypercapnia, acidosis) and decreases the need for intubation [49,50,51,52]. In addition, a meta-analysis of 13 trials found that patients with CPE who received NIV plus standard care had a lower hospital mortality than those who received standard care alone [53,54]. However, in the same analysis, treatment with NIV was only associated with a trend toward improved mortality that did not reach statistical significance. In addition, NIV did not have any impact on hospital length of stay of these patients.

A randomized trial comparing bilevel NIV with CPAP found no differences in mortality or in the incidence of intubations despite patients’ respiratory distress appearing to improve more quickly when receiving bilevel NIV [55,56,57]. Based on this evidence, ERS/ATS guidelines recommended either bilevel NIV or CPAP for patients with ARF due to CPE (strong recommendation, moderate certainty of evidence) [47].

In clinical practice, for patients with CPE, a trial of NIV, generally with CPAP, is recommended as initial therapy. Bilevel NIV may be particularly beneficial in patients with hypercapnia.

Patients with ARF should be intubated for invasive mechanical ventilation if they fail to improve with NIV (within two hours) or they do not tolerate NIV.

### 5.2. Diuretic Therapy

Intravenous loop diuretics (i.e., furosemide) are considered the cornerstone of CPE treatment. Patients with CPE are usually volume overloaded. Consequently, fluid removal with intravenous diuretics can improve symptoms and oxygenation, even in the less typical scenario where CPE occurs without severe volume overload (e.g., with hypertensive emergency, acute aortic or mitral valvular insufficiency). Although the safety and efficacy of diuretic therapy have not been proved in randomized trials, extensive observational experience has shown that they are useful for the successful treatment of most CPE patients [58]. In addition, starting intravenous diuretic treatment may also enhance hospital outcomes [10].

Exceptions in which some delay in diuretics use may be required include patients with severe hypotension or cardiogenic shock. Such patients may not have adequate LV filling pressures, and an increased diuresis may have a negative effect. On the other hand, diuresis should be increased carefully in patients with aortic stenosis and volume overload.

By reducing intravascular volume, diuresis lowers central venous and pulmonary capillary wedge pressures. In addition, furosemide also has an initial morphine-like effect that causes vasodilation and can decrease lung congestion before the onset of diuresis [59]. Intravenous rather than oral administration is recommended because of greater and more consistent drug bioavailability. The doses of furosemide should be individualized according to patient status and response [60]. Ultrafiltration (UF) or inotropes should be taken into consideration if significant congestion continues and appropriate diuresis cannot be accomplished.

### 5.3. Ultrafiltration

UF is a fluid removal procedure that is particularly useful in patients with renal dysfunction and diuretic resistance. A randomized trial demonstrated that UF was superior to the use of IV diuretics in controlling net fluid loss and rehospitalization in hypervolemic patients with HF [61]. These findings suggest that UF should be considered in patients with volume overload and CPE who have not responded well to moderate to high doses of diuretic therapy or in whom the side effects of such treatment (e.g., renal dysfunction) do not allow initiation of diuretics or make them ineffective.

### 5.4. Vasodilator Therapy

In patients with CPE, vasodilators can be needed to reduce high filling pressures and/or LV afterload. However, the routine use of vasodilators in these patients should be avoided because it does not improve the outcome [62].

The selection of a vasodilator depends on the underlying hemodynamics. For patients who do not respond well to diuretics, a vasodilator therapy that primarily lowers venous tone (such as nitroglycerin) may be administered as an addition to diuretic therapy. For individuals who require afterload reduction, a vasodilator that lowers arterial tone (such as nitroprusside) is advised (i.e., severe hypertension, acute mitral regurgitation, or acute aortic regurgitation).

The dose of vasodilators should be adjusted according to the patient’s response. In patients with right ventricular infarction or aortic stenosis, these medications should be avoided or used with caution. They should also be lowered or stopped entirely if symptomatic hypotension develops.

### 5.5. Management of Arrhythmias

Atrial fibrillation (AF) is a common arrhythmia, particularly in patients with HF. It is often difficult to determine whether AF is the cause or result of HF. Acute HF can precipitate AF due to increases in left atrial pressure and wall stress. AF can cause acute HF, particularly if the ventricular response is rapid. On the other hand, AF may be chronic and not directly related to acute HF decompensation. The treatment of AF depends upon whether or not it is associated with significant hemodynamic instability and whether or not it is believed to be the precipitant of HF decompensation. AF management starts with rate control and early decision-making regarding the need for cardioversion. If we decide to perform emergency cardioversion, the risk of a thromboembolic event needs to be considered. If cardioversion occurs within 48 h of the onset of AF, the thromboembolic risk appears to be very low. Anticoagulation prior to cardioversion (e.g., intravenous heparin) is mandatory for patients in whom cardioversion will take place more than 48 h after the onset of AF or when the duration is unknown.

In patients with preserved LVEF, rate control can be achieved with beta-blockers. In patients who cannot receive a beta blocker or are not adequately rate controlled despite their maximal-tolerated dose, digoxin may be considered as well as amiodarone, which is easier to handle although with a higher risk of returning to sinus rhythm even in those patients who should be anticoagulated [61,62,63].

### 5.6. Management of Hypotensive Patients

Patients with CPE and hypotension deserve special consideration. The goal of the therapy is to maintain systemic perfusion and preserve end-organ performance. The therapeutic approach differs for HF with reduced ejection fraction (i.e., ≤40 percent) and HF with preserved ejection fraction.

In patients with reduced ejection fraction, the administration of an inotrope (i.e., dobutamine and/or milrinone) is suggested in case of systolic blood pressure < 85–90 mmHg or evidence of shock (e.g., cool extremities, narrow pulse pressure, low urine output, confusion) and evidence of adequate preload [10,64]. The use of epinephrine or levosimendan as an alternative to dobutamine and milrinone is controversial. The use of epinephrine in the treatment of cardiogenic shock has been associated with detrimental outcomes [65,66]. Levosimendan is a calcium sensitizer that increases contractility without raising myocardial oxygen demand; it is not an arrhythmogenic agent, and it also increases coronary flow reserve. However, a randomized clinical study—the Survival of Patients With Acute Heart Failure in Need of Intravenous Inotropic Support (SURVIVE) trial—demonstrated no mortality benefit from levosimendan in comparison with dobutamine in patients with acute decompensated HF [67,68].

Due to their negative inotropic impact, beta-blockers should be stopped in patients with CPE and hypotension. In the same way, ivabradine should not be used to treat tachycardia caused by cardiogenic shock. Since milrinone and levosimendan do not act via beta receptors, their effects are not as diminished as those of dobutamine by concomitant beta-blocker therapy.

In case of persistent shock, a vasopressor (i.e., norepinephrine) may be needed as a temporizing measure to support perfusion to vital organs, though this is at the expense of increased LV afterload. Invasive monitoring, including a pulmonary artery catheter, may be useful in these patients.

Patients with preserved ejection fraction presenting with hypotension and evidence of adequate preload require a vasopressor and should not receive inotropic therapy. Assessment of the intravascular volume status is critical in such patients to determine the need for diuresis and/or hydration.

For patients who develop hypotension with dynamic LV outflow obstruction (e.g., some patients with hypertrophic cardiomyopathy), treatment may include a beta blocker, a vasopressor (i.e., phenylephrine or norepinephrine), and gentle hydration.

For selected patients with acute severe hemodynamic compromise despite adequate pharmacologic therapy with vasopressor and inotropes, nondurable mechanical support (e.g., intra-aortic balloon pump (IABP), extracorporeal circulatory membrane oxygenator (ECMO), or extracorporeal ventricular assist devices) is an option as a “bridge to decision” or “bridge to recovery” [10].

### 5.7. Additional Management Strategies

Considerations related to acute barrier disruption, which may cause more fluid formation and alveolar collapse, can lead to additional management strategies, including beta-agonists and pentoxifylline [9]. Increased levels of surfactant protein B in the circulation could help to identify patients with CPE at risk for poor responses to medical therapy due to mechanical injury to the alveoli–capillary barrier [12].

Short-acting beta-2 adrenergic agonists (e.g., albuterol) have been studied as a possible therapeutic intervention in ARDS and in stable HF patients. Theoretically, beta-agonists may improve fluid clearance, reduce lung vascular permeability, increase surfactant secretion, and have anti-inflammatory effects. Lee et al. have summarized the mixed results of studies treating beta-2 adrenergic agonists patients with ARDS [67]. Some studies have reported improvements in edema resolution using beta-2 agonists, but others found that this treatment may result in further tissue damage. Beta-2 agonists did not improve outcomes in patients with ARDS. More recent studies have found that albuterol can safely reduce extravascular lung fluid in stable HF patients without complications [69].

The principal mechanisms by which albuterol reduces lung fluid are increased lymphatic drainage, the activation of epithelial sodium channels on type I and II alveolar cells, and enhanced lymphatic drainage [69]. However, beta-2 agonists have not yet been studied in patients with CPE [9].

A recent meta-analysis of pentoxifylline administered following the diagnosis of HF reported a 4-fold decrease in all-cause mortality from 18.3% to 5.4% [70]. The good impact on patient outcomes was originally related to blocking the synthesis of TNF-alpha, but a more recent review suggests that the drug’s other immunomodulatory properties explain its benefit [14,71]. This argument is supported by a study in which idiopathic-dilated cardiomyopathy patients with elevated baseline CRP levels were more likely to respond to pentoxifylline [13]. However, more studies investigating the role of pentoxifylline in reducing pulmonary damage in patients with CPE are needed [9].

Recently, sodium-glucose co-transporter 2 inhibitors (SGLT2i) have been introduced in the treatment of heart failure. Dapagliflozin and empagliflozin reduce the risk of HF hospitalization and cardiovascular (CV) death in HF. Their true mechanism of action in HF is not yet defined. SGLT2i have earned their place as the fourth pillar of HF medical therapy alongside sacubitril-valsartan, evidence-based beta-blockers, and mineralocorticoid receptor antagonists. They should be considered for the treatment of all symptomatic patients across the entire range of HF phenotypes, including patients hospitalized with HF. Vericiguat benefits high-risk patients with worsening HF clinical profile. Most patients hospitalized with HF can be up-titrated to high doses of GDMT within weeks, and this approach reduces the likelihood of adverse HF outcomes [70,71,72].

## 6. Conclusions

Among patients, diseases causing pulmonary edema are among the most frequent causes of respiratory failure and should be treated quickly. A faster method to recognize and treat acute respiratory failure in an emergency department is a single breath counting test [73].

Moreover, chest trauma must also be treated quickly and acutely because it can cause or aggravate pulmonary edema. Certainly, chest wall blocks are revolutionizing emergency medical practice [74].

Pleuroparenchimal patterns in ALI/ARDS do find a characterization through ultrasonographic lung scans. In the critically ill, the ultrasound demonstration of a dyshomogeneous AIS with spared areas, pleural line modifications, and lung consolidations is strongly predictive, in an early phase, of non-cardiogenic pulmonary edema [75].

Patients with CPE represent a heterogeneous population. Appropriate therapy for these patients requires appropriate identification of the specific CPE phenotype as well as the potential causes and precipitants. NIV and diuretics should be considered early. Hypoperfusion requires treatment with inotropes and occasionally vasopressors. Patients with persistent symptoms and diuretic resistance might benefit from vasodilators and additional approaches to management, like beta-agonists. More studies investigating the role of medical therapy in reducing pulmonary damage in patients with CPE are needed [76,77,78].

## Figures and Tables

**Table 1:** Cancer treatments may cause cardiomyopathy.

✓ **Anthracyclines chemotherapies**: doxorubicin (Adriamycin^®^ Doxil ^®^ and Rubex^®^), daunorubicin (Cerubidine^®^), epirubicin (Ellence^®^), Valrubicin (Valstar^®^), idarubicin (Idamycin^®^) ✓ **Other chemotherapy drugs:** Trastuzumab (Herceptin^®^) Mitoxantrone (Novatrone^®^), tucatinib (Tukysa^®^) ✓ **Cisplatin** (Platinol^®^) can cause severe high blood pressure, which can lead to heart impairment. ✓ **Radiation therapy to the chest: a combination of radiation therapy and chemotherapy can increase this risk**

**Table 2:** Common and uncommon precipitating factors associated with cardiogenic pulmonary edema.

✓ **Uncontrolled hypertension** ✓ **Myocardial ischemia** ✓ **Arrhythmias** (i.e., atrial fibrillation with a rapid ventricular response, ventricular tachycardia) ✓ **Systemic infection** (especially pulmonary infection) ✓ **Severe emotional or physical stress** ✓ **Vigorous intravenous fluid administration** ✓ **Worsening renal failure** ✓ **Severe anemia** ✓ **Noncompliance with medications** (e.g., diuretics) ✓ **Thyrotoxicosis** ✓ **Medications that reduce contractility** (i.e., antiarrhythmic drugs, calcium channel blockers, beta-adrenergic blocking agents, anthracyclines, and other chemotherapeutic agents) ✓ **Cardiac toxins** (i.e., alcohol, cocaine) ✓ **Use of sodium retaining medications** (anti-inflammatory drugs, steroids) ✓ **Excessive salt or water intake**

**Table 3:** Common causes of new-onset cardiogenic pulmonary edema.

✓ **Coronary artery disease** (i.e., myocardial infarction, unstable angina) ✓ **Arrhythmias** (i.e., atrial fibrillation, atrial flutter, ventricular tachycardia) ✓ **Myocarditis** ✓ **Non-cardiac infections** (they may cause fever, which leads to tachycardia) ✓ **Acute valve syndromes** (i.e., acute mitral or aortic regurgitation secondary to infective endocarditis, ischemic papillary muscle rupture, or aortic dissection, thrombosed mechanical aortic or mitral valve, tear or perforation of a bioprosthetic aortic or mitral valve leaflet) ✓ **Ventricular septal rupture** ✓ **Stress(takotsubo) cardiomyopathy** ✓ **Volume overload** (e.g., in renal failure) ✓ **Hypertensive crisis**

**Table 4:** Common radiological findings in patients with cardiogenic pulmonary edema.

✓ Cephalization of pulmonary vessels (i.e., pulmonary venous redistribution to the upper lobes) correlates with a pulmonary capillary hydrostatic pressure greater than 12 mmHg
✓ Broad vascular pedicle (i.e., the width of the mediastinum at the level of the superior vena cava on the right and the left subclavian artery origin on the left.) correlates with a pulmonary capillary hydrostatic pressure greater than 12 mmHg
✓ Septal lines, also known as Kerley lines, are due to edema of the connective tissues of the interlobular septa and usually occur when pulmonary capillary hydrostatic reaches 17 mmHg
✓ Batwing edema (i.e., central, nongravitational distribution of alveolar edema with lung cortex free of alveolar or interstitial fluid). It correlates with pulmonary capillary hydrostatic greater than 25 mmHg.
✓ Pleural effusions and cardiomegaly (i.e., an increase of cardiothoracic ratio over 50%) are other chest x-ray findings useful for differentiating CPE from NCPE. Of note, pleural effusions and enlarged cardiac silhouette are frequently present in patients with acute decompensation of chronic HF but are often absent in patients with de novo acute HF.
✓ Unilateral cardiogenic pulmonary edema is infrequent (2% of CPE cases in one study) and is chiefly caused by eccentric mitral regurgitation

Legend: CPE, cardiogenic pulmonary edema; NCPE, non-cardiogenic pulmonary edema; HF, heart failure.

**Table 5:** Initial treatment of cardiogenic pulmonary edema.

✓ Supplemental oxygen and/or non-invasive ventilatory support to assure adequate oxygenation in case of hypoxemia
✓ Early intravenous loop diuretics (i.e., furosemide) therapy is considered the cornerstone of CPE in order to decrease cardiac preload; the aggressiveness of diuretic therapy depends on the patient’s hemodynamic and volume status
✓ Vasodilator therapy when the initial response to diuretics is not sufficient to decrease cardiac preload (e.g., nitroglycerin). Early intravenous vasodilator therapy with an agent that lowers arterial tone (e.g., nitroprusside) is suggested in patients who require a rapid decrease in cardiac afterload (e.g., those with severe hypertension, acute mitral regurgitation, acute aortic regurgitation).
✓ Management of Atrial fibrillation (AF). AF management starts with rate control (<110/min) and early decision-making regarding the need for cardioversion. Anticoagulation prior to cardioversion (e.g., intravenous heparin) is mandatory for patients in whom cardioversion will take place more than 48 h after the onset of AF or when the duration is unknown. In patients with preserved ejection fraction, rate control can be achieved with beta-blockers. In patients who cannot receive a beta blocker, digoxin may be considered.
✓ Management of hypotensive patient ◦ In patients with reduced ejection fraction, the use of an inotrope (i.e., dobutamine or milrinone) is suggested in the presence of adequate preload. Beta-blockers should be discontinued due to their negative inotropic effect; of note, milrinone does not act via beta receptors; as a consequence, its effects are not as diminished as those of dobutamine by concomitant beta-blocker therapy). In case of persistent shock, a vasopressor (i.e., phenylephrine or norepinephrine) may be needed ◦ Patients with preserved ejection fraction may require a vasopressor and should not receive inotropic therapy. Assessment of the intravascular volume status is critical in such patients to determine the need for hydration. For patients with dynamic left ventricular outflow obstruction (e.g., some patients with hypertrophic cardiomyopathy), treatment may also include beta-blockers.
✓ Venous thromboembolism prophylaxis
✓ Sodium and fluid restriction

## Data Availability

Not applicable.

## References

[1] Gheorghiade M., Zannad F., Sopko G., Klein L., Piña I.L., Konstam M.A., Massie B.M., Roland E., Targum S., Collins S.P. (2005). International Working Group on Acute Heart Failure Syndromes. Acute heart failure syndromes: Current state and framework for future research. Circulation.

[2] Nieminen M.S., Brutsaert D., Dickstein K., Drexler H., Follath F., Harjola V.P., Hochadel M., Komajda M., Lassus J., Lopez-Sendon J.L. (2006). EuroHeart Survey Investigators; Heart Failure Association, European Society of Cardiology. EuroHeart Failure Survey II (EHFS II): A survey on hospitalized acute heart failure patients: Description of population. Eur. Heart J..

[3] Crane S.D. (2002). Epidemiology, treatment and outcome of acidotic, acute, cardiogenic pulmonary oedema presenting to an emergency department. Eur. J. Emerg. Med..

[4] Wiener R.S., Moses H.W., Richeson J.F., Gatewood R.P. (1987). Hospital and long-term survival of patients with acute pulmonary edema associated with coronary artery disease. Am. J. Cardiol..

[5] Ware L.B., Matthay M.A. (2005). Clinical practice. Acute pulmonary edema. N. Engl. J. Med..

[6] Rocca E., Zanza C., Longhitano Y., Piccolella F., Romenskaya T., Racca F., Savioli G., Saviano A., Piccioni A., Mongodi S. (2023). Lung Ultrasound in Critical Care and Emergency Medicine: Clinical Review. Adv. Respir. Med..

[7] Gri N., Longhitano Y., Zanza C., Monticone V., Fuschi D., Piccioni A., Bellou A., Esposito C., Ceresa I.F., Savioli G. (2023). Acute Oncologic Complications: Clinical-Therapeutic Management in Critical Care and Emergency Departments. Curr. Oncol..

[8] Zanza C., Facelli V., Romenskaya T., Bottinelli M., Caputo G., Piccioni A., Franceschi F., Saviano A., Ojetti V., Savioli G. (2022). Lactic Acidosis Related to Pharmacotherapy and Human Diseases. Pharmaceuticals.

[9] Dobbe L., Rahman R., Elmassry M., Paz P., Nugent K. (2019). Cardiogenic Pulmonary Edema. Am. J. Med. Sci..

[10] Yancy C.W., Jessup M., Bozkurt B., Butler J., Casey D.E., Drazner M.H., Fonarow G.C., Geraci S.A., Horwich T., Januzzi J.L. (2013). American College of Cardiology Foundation/American Heart Association Task Force on Practice Guidelines. 2013 ACCF/AHA guideline for the management of heart failure: A report of the American College of Cardiology Foundation/American Heart Association Task Force on practice guidelines. Circulation.

[11] Rochwerg B., Brochard L., Elliott M.W., Hess D., Hill N.S., Nava S., Navalesi P., Antonelli M., Brozek J., Conti G. (2017). Official ERS/ATS clinical practice guidelines: Noninvasive ventilation for acute respiratory failure. Eur. Respir. J..

[12] De Pasquale C.G., Arnolda L.F., Doyle I.R., Grant R.L., Aylward P.E., Bersten A.D. (2003). Prolonged alveolocapillary barrier damage after acute cardiogenic pulmonary edema. Crit. Care Med..

[13] Sliwa K., Woodiwiss A., Libhaber E., Zhanje F., Libhaber C., Motara R., Essop R. (2004). C-reactive protein predicts response to pentoxifylline in patients with idiopathic dilated cardiomyopathy. Eur. J. Heart Fail..

[14] Shaw S.M., Shah M.K., Williams S.G., Fildes J.E. (2009). Immunological mechanisms of pentoxifylline in chronic heart failure. Eur. J. Heart Fail..

[15] Herrero R., Sanchez G. (2018). New insights into the mechanisms of pulmonary edema in acute lung injury. Ann. Transl. Med..

[16] Iles K.E., Song W., Miller D.W., Dickinson D.A., Matalon S. (2009). Reactive species and pulmonary edema. Expert Rev. Respir. Med..

[17] Racca F., Longhitano Y., Zanza C., Draisci G., Stoia P.A., Gollo E., Maio M., Grattarola C., Astuto M., Vaschetto R. (2023). Peri-Partum respiratory management in neuro-muscular disorders (IT-NEUMA-Pregn study): A proposal by an italian panel and a call for an international collaboration. Pulmonology.

[18] Murray J.F. (2011). Pulmonary edema: Pathophysiology and diagnosis. Int. J. Tuberc. Lung Dis..

[19] Szidon J.P. (1989). Pathophysiology of the congested lung. Cardiol. Clin..

[20] Gropper M.A., Wiener-Kronish J.P., Hashimoto S. (1994). Acute cardiogenic pulmonary edema. Clin. Chest Med..

[21] Günther A., Siebert C., Schmidt R., Ziegler S., Grimminger F., Yabut M., Temmesfeld B., Walmrath D., Morr H., Seeger W. (1996). Surfactant alterations in severe pneumonia, acute respiratory distress syndrome, and cardiogenic lung edema. Am. J. Respir. Crit. Care Med..

[22] Wang H.C., Zentner M.D., Deng H.T., Kim K.J., Wu R., Yang P.C., Ann D.K. (2000). Oxidative stress disrupts glucocorticoid hormone-dependent transcription of the amiloride-sensitive epithelial sodium channel α-subunit in lung epithelial cells through ERK-dependent and thioredoxin-sensitive pathways. J. Biol. Chem..

[23] Matthay M.A., Ware L.B., Zimmerman G.A. (2012). The acute respiratory distress syndrome. J. Clin. Investig..

[24] Zemans R.L., Matthay M.A. (2004). Bench-to-bedside review: The role of the alveolar epithelium in the resolution of pulmonary edema in acute lung injury. Crit. Care.

[25] Vivona M.L., Matthay M., Chabaud M.B., Friedlander G., Clerici C. (2001). Hypoxia reduces alveolar epithelial sodium and fluid transport in rats: Reversal by β-adrenergic agonist treatment. Am. J. Respir. Cell Mol. Biol..

[26] Roux J., Kawakatsu H., Gartland B., Pespeni M., Sheppard D., Matthay M.A., Canessa C.M., Pittet J.F. (2005). Interleukin-1β decreases expression of the epithelial sodium channel α-subunit in alveolar epithelial cells via a p38 MAPK-dependent signaling pathway. J. Biol. Chem..

[27] Herrero R., Kajikawa O., Matute-Bello G., Wang Y., Hagimoto N., Mongovin S., Wong V., Park D.R., Brot N., Heinecke J.W. (2011). The biological activity of FasL in human and mouse lungs is determined by the structure of its stalk region. J. Clin. Investig..

[28] Herrero R., Tanino M., Smith L.S., Kajikawa O., Wong V.A., Mongovin S., Matute-Bello G., Martin T.R. (2013). The Fas/FasL pathway impairs the alveolar fluid clearance in mouse lungs. Am. J. Physiol. Lung Cell. Mol. Physiol..

[29] Perl M., Lomas-Neira J., Chung C.-S., Ayala A. (2008). Epithelial cell apoptosis and neutrophil recruitment in acute lung injury-a unifying hypothesis? What we have learned from small interfering RNAs. Mol. Med..

[30] Elia N., Tapponnier M., Matthay M.A., Hamacher J., Pache J.-C., Bründler M.-A., Totsch M., De Baetselier P., Fransen L., Fukuda N. (2003). Functional identification of the alveolar edema reabsorption activity of murine tumor necrosis factor-α. Am. J. Respir. Crit. Care Med..

[31] Dagenais A., Fréchette R., Yamagata Y., Yamagata T., Carmel J.-F., Clermont M.-E., Brochiero E., Massé C., Berthiaume Y. (2004). Downregulation of ENaC activity and expression by TNF-α in alveolar epithelial cells. Am. J. Physiol. Lung Cell. Mol. Physiol..

[32] Lee K.S., Choi Y.H., Kim Y.S., Baik S.H., Oh Y.J., Sheen S.S., Park J.H., Hwang S.C., Park K.J. (2008). Evaluation of bronchoalveolar lavage fluid from ARDS patients with regard to apoptosis. Respir. Med..

[33] Alwi I. (2010). Diagnosis and management of cardiogenic pulmonary edema. Acta Medica Indones..

[34] Piper S.E., McDonagh T.A. (2015). Chemotherapy-related Cardiomyopathy. Eur. Cardiol..

[35] McDonagh T.A., Metra M., Adamo M., Gardner R.S., Baumbach A., Böhm M., Burri H., Butler J., Čelutkienė J., Chioncel O. (2021). ESC Scientific Document Group. 2021 ESC Guidelines for the diagnosis and treatment of acute and chronic heart failure. Eur. Heart J..

[36] Al Deeb M., Barbic S., Featherstone R., Dankoff J., Barbic D. (2014). Point-of-care ultrasonography for the diagnosis of acute cardiogenic pulmonary edema in patients presenting with acute dyspnea: A systematic review and meta-analysis. Acad. Emerg. Med..

[37] Thomas S.S., Nohria A. (2012). Hemodynamic classifications of acute heart failure and their clinical application—An update. Circ. J..

[38] Gluecker T., Capasso P., Schnyder P., Gudinchet F., Schaller M.D., Revelly J.P., Chiolero R., Vock P., Wicky S. (1999). Clinical and radiologic features of pulmonary edema. Radiographics.

[39] Attias D., Mansencal N., Auvert B., Vieillard-Baron A., Delos A., Lacombe P., N’Guetta R., Jardin F., Dubourg O. (2010). Prevalence, characteristics, and outcomes of patients presenting with cardiogenic unilateral pulmonary edema. Circulation.

[40] Agricola E., Bove T., Oppizzi M., Marino G., Zangrillo A., Margonato A., Picano E. (2005). “Ultrasound comet-tail images”: A marker of pulmonary edema: A comparative study with wedge pressure and extravascular lung water. Chest.

[41] Coiro S., Porot G., Rossignol P., Ambrosio G., Carluccio E., Tritto I., Huttin O., Lemoine S., Sadoul N., Donal E. (2016). Prognostic value of pulmonary congestion assessed by lung ultrasound imaging during heart failure hospitalisation: A two-centre cohort study. Sci. Rep..

[42] Volpicelli G., Elbarbary M., Blaivas M., Lichtenstein D.A., Mathis G., Kirkpatrick A.W., Melniker L., Gargani L., Noble V.E., Via G. (2012). International Liaison Committee on Lung Ultrasound (ILC-LUS) for International Consensus Conference on Lung Ultrasound (ICC-LUS). International evidence-based recommendations for point-of-care lung ultrasound. Intensive Care Med..

[43] Maisel A.S., Krishnaswamy P., Nowak R.M., McCord J., Hollander J.E., Duc P., Omland T., Storrow A.B., Abraham W.T., Wu A.H. (2002). Breathing Not Properly Multinational Study Investigators. Rapid measurement of B-type natriuretic peptide in the emergency diagnosis of heart failure. N. Engl. J. Med..

[44] Lindenfeld J., Albert N.M., Boehmer J.P., Collins S.P., Ezekowitz J.A., Givertz M.M., Katz S.D., Klapholz M., Moser D.K., Heart Failure Society of America (2010). HFSA 2010 Comprehensive Heart Failure Practice Guideline. J. Card. Fail..

[45] Hunt S.A., Abraham W.T., Chin M.H., Feldman A.M., Francis G.S., Ganiats T.G., Jessup M., Konstam M.A., Mancini D.M., Michl K. (2009). 2009 focused update incorporated into the ACC/AHA 2005 Guidelines for the Diagnosis and Management of Heart Failure in Adults: A report of the American College of Cardiology Foundation/American Heart Association Task Force on Practice Guidelines: Developed in collaboration with the International Society for Heart and Lung Transplantation. Circulation.

[46] Park J.H., Balmain S., Berry C., Morton J.J., McMurray J.J. (2010). Potentially detrimental cardiovascular effects of oxygen in patients with chronic left ventricular systolic dysfunction. Heart.

[47] Racca F., Geraci C., Cremascoli L., Ruvolo D., Piccolella F., Romenskaya T., Longhitano Y., Martuscelli E., Saviano A., Savioli G. (2023). Invasive Mechanical Ventilation in Traumatic Brain Injured Patients with Acute Respiratory Failure. Rev. Recent Clin. Trials..

[48] Williams J.W., Cox C.E., Hargett C.W., Gilstrap D.L., Castillo C.E., Govert J.A., Lugogo N.L., Coeytaux R.R., McCrory D.C., Hasselblad V. (2012). Noninvasive Positive-Pressure Ventilation (NPPV) for Acute Respiratory Failure [Internet].

[49] Masip J., Roque M., Sánchez B., Fernández R., Subirana M., Expósito J.A. (2005). Noninvasive ventilation in acute cardiogenic pulmonary edema: Systematic review and meta-analysis. JAMA.

[50] Winck J.C., Azevedo L.F., Costa-Pereira A., Antonelli M., Wyatt J.C. (2006). Efficacy and safety of non-invasive ventilation in the treatment of acute cardiogenic pulmonary edema--a systematic review and meta-analysis. Crit. Care.

[51] Collins S.P., Mielniczuk L.M., Whittingham H.A., Boseley M.E., Schramm D.R., Storrow A.B. (2006). The use of noninvasive ventilation in emergency department patients with acute cardiogenic pulmonary edema: A systematic review. Ann. Emerg. Med..

[52] Gray A., Goodacre S., Newby D.E., Masson M., Sampson F., Nicholl J., 3CPO Trialists (2008). Noninvasive ventilation in acute cardiogenic pulmonary edema. N. Engl. J. Med..

[53] Weng C.L., Zhao Y.T., Liu Q.H., Fu C.J., Sun F., Ma Y.L., Chen Y.W., He Q.Y. (2010). Meta-analysis: Noninvasive ventilation in acute cardiogenic pulmonary edema. Ann. Intern. Med..

[54] Zanza C., Longhitano Y., Leo M., Romenskaya T., Franceschi F., Piccioni A., Pabon I.M., Santarelli M.T., Racca F. (2022). Practical Review of Mechanical Ventilation in Adults and Children in the Operating Room and Emergency Department. Rev. Recent Clin. Trials.

[55] Vital F.M., Ladeira M.T., Atallah A.N. (2013). Non-invasive positive pressure ventilation (CPAP or bilevel NPPV) for cardiogenic pulmonary oedema. Cochrane Database Syst. Rev..

[56] Rusterholtz T., Kempf J., Berton C., Gayol S., Tournoud C., Zaehringer M., Jaeger A., Sauder P. (1999). Noninvasive pressure support ventilation (NIPSV) with face mask in patients with acute cardiogenic pulmonary edema (ACPE). Intensive Care Med..

[57] Liesching T., Nelson D.L., Cormier K.L., Sucov A., Short K., Warburton R., Hill N.S. (2014). Randomized trial of bilevel versus continuous positive airway pressure for acute pulmonary edema. J. Emerg. Med..

[58] Felker G.M., O’Connor C.M., Braunwald E. (2009). Heart Failure Clinical Research Network Investigators. Loop diuretics in acute decompensated heart failure: Necessary? Evil? A necessary evil?. Circ. Heart Fail..

[59] Dikshit K., Vyden J.K., Forrester J.S., Chatterjee K., Prakash R., Swan H.J. (1973). Renal and extrarenal hemodynamic effects of furosemide in congestive heart failure after acute myocardial infarction. N. Engl. J. Med..

[60] Felker G.M., Lee K.L., Bull D.A., Redfield M.M., Stevenson L.W., Goldsmith S.R., LeWinter M.M., Deswal A., Rouleau J.L., Ofili E.O. (2011). NHLBI Heart Failure Clinical Research Network. Diuretic strategies in patients with acute decompensated heart failure. N. Engl. J. Med..

[61] Costanzo M.R., Guglin M.E., Saltzberg M.T., Jessup M.L., Bart B.A., Teerlink J.R., Jaski B.E., Fang J.C., Feller E.D., Haas G.J. (2007). UNLOAD Trial Investigators. Ultrafiltration versus intravenous diuretics for patients hospitalized for acute decompensated heart failure. J. Am. Coll. Cardiol..

[62] National Clinical Guideline Centre (UK) (2014). Acute Heart Failure: Diagnosing and Managing Acute Heart Failure in Adults.

[63] Hofmann R., Steinwender C., Kammler J., Kypta A., Wimmer G., Leisch F. (2004). Intravenous amiodarone bolus for treatment of atrial fibrillation in patients with advanced congestive heart failure or cardiogenic shock. Wien. Klin. Wochenschr..

[64] McMurray J.J., Adamopoulos S., Anker S.D., Auricchio A., Böhm M., Dickstein K., Falk V., Filippatos G., Fonseca C., Gomez-Sanchez M.A. (2012). ESC Committee for Practice Guidelines. ESC Guidelines for the diagnosis and treatment of acute and chronic heart failure 2012: The Task Force for the Diagnosis and Treatment of Acute and Chronic Heart Failure 2012 of the European Society of Cardiology. Developed in collaboration with the Heart Failure Association (HFA) of the ESC. Eur. Heart J..

[65] Tarvasmäki T., Lassus J., Varpula M., Sionis A., Sund R., Køber L., Spinar J., Parissis J., Banaszewski M., Silva Cardoso J. (2016). CardShock study investigators. Current real-life use of vasopressors and inotropes in cardiogenic shock—Adrenaline use is associated with excess organ injury and mortality. Crit. Care.

[66] Léopold V., Gayat E., Pirracchio R., Spinar J., Parenica J., Tarvasmäki T., Lassus J., Harjola V.P., Champion S., Zannad F. (2018). Epinephrine and short-term survival in cardiogenic shock: An individual data meta-analysis of 2583 patients. Intensive Care Med..

[67] Mebazaa A., Nieminen M.S., Packer M., Cohen-Solal A., Kleber F.X., Pocock S.J., Thakkar R., Padley R.J., Põder P., Kivikko M. (2007). SURVIVE Investigators. Levosimendan vs dobutamine for patients with acute decompensated heart failure: The SURVIVE Randomized Trial. JAMA.

[68] Lee J.W. (2009). beta2 adrenergic agonists in acute lung injury? The heart of the matter. Crit. Care.

[69] Taylor B.J., Snyder E.M., Richert M.L., Wheatley C.M., Chase S.C., Olson L.J., Johnson B.D. (2017). Effect of β2-adrenergic receptor stimulation on lung fluid in stable heart failure patients. J. Heart Lung Transplant..

[70] Velez M. (2023). Advances in contemporary medical management to treat patients with heart failure. Curr. Opin. Cardiol..

[71] Cox Z.L., Collins S.P., Aaron M., Hernandez G.A., Davidson B.T., Fowler M., Lindsell C.J., Frank E.H., A Jenkins C.A. (2021). Efficacy and safety of dapagliflozin in acute heart failure: Rationale and design of the DICTATE-AHF trial. Am. Heart J..

[72] Carballo S., Stirnemann J., Garin N., Darbellay Farhoumand P., Serratrice J., Carballo D. (2020). Prognosis of patients eligible for dapagliflozin in acute heart failure. Eur. J. Clin. Investig..

[73] Longhitano Y., Zanza C., Romenskaya T., Saviano A., Persiano T., Leo M., Piccioni A., Betti M., Maconi A., Pindinello I. (2021). Single-Breath Counting Test Predicts Non-Invasive Respiratory Support Requirements in Patients with COVID-19 Pneumonia. J. Clin. Med..

[74] Zanza C., Romenskaya T., Zuliani M., Piccolella F., Bottinelli M., Caputo G., Rocca E., Maconi A., Savioli G., Longhitano Y. (2023). Acute Traumatic Pain in the Emergency Department. Diseases.

[75] Copetti R., Soldati G., Copetti P. (2008). Chest sonography: A useful tool to differentiate acute cardiogenic pulmonary edema from acute respiratory distress syndrome. Cardiovasc. Ultrasound.

[76] Savioli G., Ceresa I.F., Manzoni F., Ricevuti G., Bressan M.A., Oddone E. (2020). Role of a Brief Intensive Observation Area with a Dedicated Team of Doctors in the Management of Acute Heart Failure Patients: A Retrospective Observational Study. Medicina.

[77] Ceresa I.F., Savioli G., Angeli V., Novelli V., Muzzi A., Grugnetti G., Cobianchi L., Manzoni F., Klersy C., Lago P. (2020). Preparing for the Maximum Emergency with a Simulation: A Table-Top Test to Evaluate Bed Surge Capacity and Staff Compliance with Training. Open Access Emerg. Med..

[78] Piccioni A., Valletta F., Franza L., Rosa F., Manca F., Zanza C., Savioli G., Gasbarrini A., Covino M., Franceschi F. (2023). Evaluation of procalcitonin in hemorrhagic shock: A pilot study. Clin. Ter..

